# Diversity of *SCCmec* elements in Staphylococci isolated from public washrooms

**DOI:** 10.1186/s12866-015-0451-3

**Published:** 2015-06-14

**Authors:** Hermine V. Mkrtchyan, Zhen Xu, Ronald R. Cutler

**Affiliations:** Pre- Clinical Drug Discovery Group, Queen Mary University of London, School of Biological and Chemical Sciences, Mile End Road, London, E1 4NS UK; Biomedical Science Degree Programmes, Pre- Clinical Drug Discovery Group, Mile End Road, London, UK

**Keywords:** Staphylococcal species, Environmental isolates, EMRSA-15

## Abstract

**Background:**

Staphylococci are commonly associated with antibiotic resistance in healthcare settings including resistance to methicillin and other beta lactam antibiotics primarily associated with the carriage of SCC*mec* mobile genetic elements. We investigated oxacillin susceptibility in 11 different species of environmental staphylococci to evaluate the potential dissemination of such resistance determinants in staphylococcal isolates from non-healthcare environments.

**Results:**

Staphylococci isolated from public washrooms were analysed for SCC*mec* type and for antibiotic susceptibility. MICs to oxacillin ranged from 0.12 to 128 mg/L. Of the 32 strains investigated, there were representatives of 11 different species of staphylococci. 21 of the 32 isolates were assigned to known SCC*mec* types. These typeable strains primarily included those assigned to SCC*mec* type I (n = 8), type IV (n = 6) and type VI (n = 7)*.* In addition to these isolates, 3 strains of EMRSA-15 were identified from different environmental sites.

**Conclusions:**

We have demonstrated the diversity of SCC*mec* elements in a wide range of staphylococcal species isolated from outside of healthcare settings. We have also shown that the variability in oxacillin MICs in such isolates are unrelated to species or SCC*mec* type. The isolation of EMRSA-15 is also of concern to infection control in the community.

## Background

The continuing spread and development of antibiotic resistance in bacteria is recognised as a major threat to healthcare worldwide [1–3]. In the past, hospital acquired MRSA (HAMRSA) were exemplified as examples of the development and spread of drug resistance in healthcare settings however the later isolation of community acquired MRSA (CAMRSA) demonstrated that antibiotic resistant *Staphylococcus aureus* were also present outside of healthcare environments. Today, increasing attention is being paid to multidrug-resistant coagulase-negative staphylococci (MRCoNS) and their spread as opportunistic pathogens, often associated with infections in immuno-compromised patients in healthcare settings [4–8].

Methicillin resistance is associated with the *mecA* gene that encodes for penicillin-binding protein PBP2a which has a low binding affinity to all β-lactam antibiotics [9]. The *mecA* gene is located on the staphylococcal cassette chromosome (SCC*mec*), a mobile genetic element with two essential components, the *mec* gene complex and the cassette chromosome recombinase (*ccr*) complex. To date, eleven (I-XI) SCC*mec* types have been fully identified by the determination of *mec* (A, B, C1, C2 and D) and *ccr* (i.e. *ccr* AB1 to *ccr*AB5 and *ccrC*) complexes [10–12].

Environmental contamination with multiply drug-resistant bacteria in healthcare settings is well documented [2, 6, 13] and it has been proposed that the development of resistance in these environments is a consequence of human activities such as antibiotic misuse and/or to mutation/DNA modifying strategies in bacteria [14]. Examples of drug resistance in staphylococci isolated from non-healthcare environments have also been reported by other workers, Soge *et al.* [15] isolated multiply drug resistant *Staphylococcus aureus* (MRSA) and MRCoNS from public beaches, Coombs *et al.* [16] reported on regional variation with CAMRSA, Simoes *et al.* [17] reported that public buses were a reservoir of EMRSA-15 and ourselves, where we identified drug resistance in a wide variety of environmental staphylococci isolated from non-healthcare public restrooms [18].

In this current study, we report on the phenotypic expression and the diversity of SCC*mec* elements in oxacillin resistant determinants found in a wide range of different staphylococcal species isolated from non-hospital environments*.*

## Results and discussion

### Identification of environmental isolates and antibiotic resistance

Thirty two staphylococcal isolates with members belonging to 11 different staphylococcal species were used in this study. In these strains MICs to oxacillin varied from 0.12/0.25 mg/L to 128 mg/L (Table 1). As reported previously by other workers the MIC breakpoint to oxacillin for staphylococci is 2 mg/L. However the MIC breakpoint for *S.epidermidis* alone has been reported to be 1 mg/L [19, 20].

**Table 1:** SCC*mec* types and Oxacillin MIC values for staphylococcal isolates

Species	No	SCCmec types	Oxacillin MICs (mg/L) (there were no isolates with MICs within 4-32 mg/L range)					
			0.12- 0.25	0.5	1	2	64	128
*S. hominis*	9	I	3					
		VI			1			
		N/T	5					
*S. aureus*	4	IV				3^a^		
		VI					1	
*S. warneri*	4	I	1					
		IV			1			
		N/T	2					
*S. epidermidis*	4	I		1				
		VI					1	1
		N/T					1	
*S. haemolyticus*	3	I	2					
		VI		1				
*S. simulans*	3	IV		1				
		N/T	1					1
*S. saprophyticus*	1	I	1					
*S. equorum*	1	VI					1	
*S. arlettae*	1	IV				1		
*S. condimeniti*	1	VI	1					
*S. cohnii*	1	N/T		1				

MIC breakpoint for staphylococci to oxacillin is 2 mg/L. MIC breakpoint for *S.epidermidis* to oxacillin is 1 mg/L [19, 20]

*N/T* non-typeable, *No* number of isolates

^a^-3 EMRSA-15 strains were determined by IVh SCC*mec* carriage, MLST and *spa* typing (Table 3)

### Molecular characterisation of environmental staphylococcal isolates

Twenty-one out of the 32 strains tested were assigned to known SCC*mec* types, 11 were untypeable. The 21 typeable strains included those assigned to type I (n = 8), type IV (n = 6) and type VI (n = 7) SCC*mec* types (Table 2, Table 3). The Class B *mec* complex was detected in all isolates, including those which were untypeable. As for *ccr* complexes, we identified 8 *ccr*AB1, 3 *ccr*AB2, and 7 *ccr*AB4 amongst these staphylococcal isolates (Table 2). SCC*mec* types for EMRSA-15 were determined using IVh primers (Table 3). In addition, we identified *S. hominis, S. warneri, S. simulans,* and *S. epidermidis* strains which carried untypeable *ccr* complexes but nevertheless harboured the *mecB complex*.

**Table 2:** SCC*mec* types of typeable staphylococci isolates determined by combination of *mec* and *ccr* complexes

*Species*	No	*mec* complex	*ccr*	SCC*mec*
*S. hominis*	3	B	1	I
*S epidermidis*	2	B	4	VI
*S. haemolyticus*	2	B	1	I
*S. arlettae*	1	B	2	IV
*S. aureus*	1	B	4	VI
*S. condimeniti*	1	B	4	VI
*S epidermidis*	1	B	1	I
*S. equorum*	1	B	4	VI
*S. haemolyticus*	1	B	4	VI
*S. hominis*	1	B	4	VI
*S. saprophyticus*	1	B	1	I
*S. simulans*	1	B	2	IV
*S. warneri*	1	B	2	IV
*S.warneri*	1	B	1	I

**Table 3:** Molecular characterization of EMRSA15 from public restrooms

*Species*	*mecA*	SCC*mec*	*spa*	MLST
*S. aureus*	+	IVh	t032	ST22
*S. aureus*	+	IVh	t032	ST22
*S. aureus*	+	IVh	t032	ST22

### MRCoNS

We found there was a wide diversity of SCC*mec* elements in a variety of different staphylococcal species isolated from non-healthcare environments, and thus diversity of SCC*mec* can be extensive. We found for example that the frequency of type I SCC*mec* elements in such staphylococcal isolates was greater than that of any of the other SCC*mec* elements. In contrast, isolates carrying SCC*mec* types V, VII, IX, X or XI were not found in this study. However in healthcare settings, many of the species we identified may not be investigated, although some workers have reported on specific MRCoNS from hospitals [6, 8]. The majority of hospital strains tested were *S. epidermidis* and *S. hominis* and in these isolates SCC*mec* types III, IV and V were the most prevalent. It has also been reported, as we have found in environmental strains, that such isolates could contain multiple types of SCC*mec* [6].

### SCC*mec* types

The association between the carriage of certain SCC*mec* types and staphylococcal species has been investigated in hospital and some SCC*mec* types have been reported to be associated with certain species. Type IV for example, was found to be primarily associated with hospital *S. epidermidis* [6]. With environmental strains however we found that SCC*mec* type IV was associated with *S. warneri, S. simulans* and *S. arlettae. S. epidermidis,* by contrast, was associated with SCC*mec* types I and VI. In addition to this, SCC*mec* type I, which requires a combination of *mec* complex B – *ccr*AB1, was prevalent in many staphylococci in our study. We found it in *S. hominis (*n = 3*), S. warneri* (n = 1)*, S. haemolyticus* (n = 2)*, S. saprophyticus* (n = 1) and *S. epidermidis* (n = 1)*.* Again, this is in contrast to previous hospital studies where it was reported that *S. hominis* isolated from clinical specimens was preferentially associated with *ccr*AB1, *ccr*AB4 and *mec* complex A [5]. These workers also described *mec-ccr* combinations in *S. hominis*. They identified SCC*mec* types I, VI, VIII and type new1, but there was a complete absence of SCC*mec* structures containing *ccr*AB2*, ccr*AB3*, ccr*C*,* and *mec* complex C. We also failed to detect *mec* complex C in any of our isolates. The environmental isolates in our study were found to be a reservoir for *mec* complex B (Table 2). Bouchami and coworkers [5] proposed that the wide genetic diversity observed for coagulase negative isolates such as *S. hominis* was associated with the presence of multiple transposases and intergrases in the staphylococcal genome, this may also be the case with environmental isolates.

### SCC*mec* carriage and MICs

Oxacillin MICs for all strains containing SCC*mec* types were determined. We found that the majority of coagulase negative strains (78.6 %) had relatively low MICs (0.12-1 mg/L), MICs for *S. aureus* strains could be high but still ranged from as low as 2 mg/L up to 64 mg/L. In addition, there were MRCoNS strains in which the SCC*mec* was untypeable which produced high MIC values to oxacillin (64–128 mg/L). This was found in 2 different species, *S. simulans* and *S. epidermidis* (Table 1). Overall, comparing our results with previously published MIC breakpoints [19, 20], we determined that 31.25 % of isolates in our study were resistant to oxacillin and 68.75 % susceptible. In contrast to our findings, in a recent study of the levels of methicillin resistance in non-healthcare associated *S.aureus* isolated from food, the authors found that strains with MICs of 2 mg/L could be either *mec*A positive or *mec*A negative and other workers have reported that mutations in the *mec* complex in *S.aureus* can also lead to variations in MICs [8, 9]. The wide variation in MICs we found suggests that such mutations may also occur in isolates taken from outside of hospital settings. No strains were identified with MICs in the range 4–32 mg/L.

### EMRSA-15 in public restrooms

*spa* typing revealed that three of the MRSA strains belonged to the internationally disseminated EMRSA-15 clone with *spa* type t032 and MLST ST22 (Table 3). These isolates were also SCC*mec* subtype IVh positive. A study by Simoes and coworkers [17] demonstrated that buses, another area heavily used by the public, were also a potential reservoir for EMRSA-15. The isolates from that study and those from this study were multiply drug resistant. More than 95 % of the MRSA bacteremia’s in the UK are with EMRSA-15 and EMRSA-16 underscoring the importance of these isolates [17].

### Persistence of antibiotic resistance genes in the environment

It is possible that a number of factors, including temperature, humidity, the presence of human faeces/waste and the misuse of antibiotics and disinfectants (where the wrong agent is used or the agent is used at an inappropriate concentration), could contribute to the survival and spread of these environmental antibiotic resistant bacteria [14]. In addition, the mobilization of antibiotic resistant elements could also play an important role in spreading resistance genes in these environments [16].

Although only thirty two isolates were used in this study, we have shown that some well-established antibiotic resistance genes persist in the environment. This is in keeping with the ideas published in a review by Martinez in 2009 [14]. In that review it was suggested that antibiotic resistance genes originally developed in non-clinical environments. In these environments, they may have acted to control metabolic processes or for cell signalling. However, the release by man of high concentrations of antibiotics into the environment and the spread of antibiotic resistance genes has impacted on the carriage of antibiotic resistance in the environment.

Previously, we reported that public restrooms are potential reservoirs of multidrug resistant staphylococci [18]. We have now demonstrated that resistance determinants show variation between environmental isolates and commonly associated hospital isolates such as *S.epidermidis* and *S.hominis* [5, 6]. Further work is needed to establish if there are any links between species isolated from hospitals and those from non-clinical environments, which affect transfer of antibiotic resistance determinants. It has been suggested that environmental bacteria may respond to subclinical levels of antibiotics in the environment and thus can induce adaptive responses with low levels of resistance in some strains [21]. Many of the environmental isolates in our study possessed low (sub-clinical) levels of resistance to oxacillin (0.12- 0.25 mg/L). These were even lower than the 0.5-4 mg/L MICs found in low/borderline level oxacillin resistant clinical isolates of *S. aureus* reported by Tomasz [22].

## Conclusions

The spread of low levels antibiotic resistance reported above has demonstrated the diversity of SCC*mec* elements in a wide range of staphylococcal species isolated from outside of healthcare settings. In addition to this, the finding of EMRSA-15 in non-healthcare environments provides further evidence that infection control measures, both in the hospitals and in public places, fails to limit the spread of such clones and once more emphasises the importance of good hygiene in these environments.

## Methods

### Bacterial isolates and susceptibility to oxacillin

Thirty two staphylococcal strains which had been isolated from 18 randomly selected public restrooms (non-healthcare) in London, United Kingdom [18] were used in this study. Dry sterile cotton swabs (Copan Diagnostics Inc., USA) were used to collect samples. Sampling was over a period of 24 weeks between December 2010 and June 2011. 21 sites were sampled in each restroom. All specimens were transferred to the laboratory within 1-3 h of the sample being taken. In the laboratory, swabs were suspended in 1 ml sterile 0.9 % saline, inoculated onto Nutrient Agar (Oxoid, Basingstoke, UK) and Mannitol Salt Agar (Oxoid, Basingstoke, UK) . Plates were incubated aerobically at 37 °C for 24-48 h to obtain pure cultures.

Staphylococcal isolates were provisionally identified using conventional methods, including microscopy, catalase and coagulase testing [18]. Species were fully identified using Matrix-assisted laser desorption ionization time flight mass-spectroscopy (MALDI-TOF-MS, Microflex LT, Bruker Daltonics, Coventry, UK) [18]. Minimum Inhibitory Concentrations (MIC) to oxacillin were evaluated using “M.I.C. evaluators” (Oxoid Ltd., Basingstoke,UK). MICs were assigned on the basis of the Guidelines for Susceptibility Testing (BSAC, 2011) [19].

### Molecular characterization of Staphylococcal isolates

#### SCCmec typing

Genomic DNA was prepared using the QIAamp DNA mini kit (Qiagen, Crawley, UK). The presence of *mecA* was determined as described previously [18]. SCC*mec* types were determined by evaluating *mec* and *ccr* complexes using primers previously described by others [23–26]. Type was determined using the primers J IVh F 5′-TTCCTCGTTTTTTCTGAACG-3′ and J IVh R 5′-CAAACACTGATATTGTGTCG-3′ as previously described [27].

SCC*mec* types were categorised according to guidelines published by the International Working Group on the Classification of *Staphylococcal* Cassette Chromosome Elements (IWG-SCC) [28] which are based on the classification of *mec* and *ccr* types. SCC*mec* was considered as untypeable if the primers used for PCR amplification of *mec* or *ccr* complexes did not yield amplicons.

#### MLST and Spa typing

*S. aureus* strains were analysed by Multilocus sequence typing (MLST) of seven housekeeping genes, as previously described by others [29]. Allele types were assigned using *S. aureus* MLST database (www.mlst.net). For *S. aureus* strains the x-region of the protein A (*spa*) gene was amplified by PCR using previously described primers [30, 31]. *spa* types were determined using the Ridom StaphType program (Ridom GmbH, Wurzburg, Germany). Sequences for each *spa* gene have been deposited in the GenBank under the accession numbers KR048283, KR048284, KR048285.

## Abbreviations

MRSA: Methicillin resistant *Staphylococcus aureus*
HAMRSA: Hospital acquired MRSA
CAMRSA: Community acquired MRSA
EMRSA: Epidemic MRSA
MRCoNS: Multi-drug resistant coagulase negative staphylococci
SCC*mec*: Staphylococci cassette chromosome
*ccr*: Cassette chromosome recombinase
MIC: Minimum inhibitory concentration
MLST: Multilocus sequence typing
PCR: Polymerase chain reaction
